# Assessment of water, sanitation and hygiene interventions in response to an outbreak of typhoid fever in Neno District, Malawi

**DOI:** 10.1371/journal.pone.0193348

**Published:** 2018-02-23

**Authors:** Sarah D. Bennett, Sara A. Lowther, Felix Chingoli, Benson Chilima, Storn Kabuluzi, Tracy L. Ayers, Thomas A. Warne, Eric Mintz

**Affiliations:** 1 Epidemic Intelligence Service, Centers for Disease Control and Prevention, Atlanta, Georgia, United States of America; 2 Division of Foodborne, Waterborne, and Environmental Diseases, Centers for Disease Control and Prevention, Atlanta, Georgia, United States of America; 3 Global Immunization Division, Centers for Disease Control and Prevention, Atlanta, Georgia, United States of America; 4 Neno District Health Office, Neno, Malawi; 5 Community Health Services Unit, Ministry of Health, Lilongwe, Malawi; 6 Division of Global HIV AIDS, Centers for Disease Control and Prevention, Lilongwe, Malawi; Public Library of Science, UNITED KINGDOM

## Abstract

On May 2, 2009 an outbreak of typhoid fever began in rural villages along the Malawi-Mozambique border resulting in 748 illnesses and 44 deaths by September 2010. Despite numerous interventions, including distribution of WaterGuard (WG) for in-home water treatment and education on its use, cases of typhoid fever continued. To inform response activities during the ongoing Typhoid outbreak information on knowledge, attitudes, and practices surrounding typhoid fever, safe water, and hygiene were necessary to plan future outbreak interventions. In September 2010, a survey was administered to female heads in randomly selected households in 17 villages in Neno District, Malawi. Stored household drinking water was tested for free chlorine residual (FCR) levels using the N,N diethyl-p-phenylene diamine colorimetric method (HACH Company, Loveland, CO, USA). Attendance at community-wide educational meetings was reported by 56% of household respondents. Respondents reported that typhoid fever is caused by poor hygiene (77%), drinking unsafe water (49%), and consuming unsafe food (25%), and that treating drinking water can prevent it (68%). WaterGuard, a chlorination solution for drinking water treatment, was observed in 112 (56%) households, among which 34% reported treating drinking water. FCR levels were adequate (FCR ≥ 0.2 mg/L) in 29 (76%) of the 38 households who reported treatment of stored water and had stored water available for testing and an observed bottle of WaterGuard in the home. Soap was observed in 154 (77%) households, among which 51% reported using soap for hand washing. Educational interventions did not reach almost one-half of target households and knowledge remains low. Despite distribution and promotion of WaterGuard and soap during the outbreak response, usage was low. Future interventions should focus on improving water, sanitation and hygiene knowledge, practices, and infrastructure. Typhoid vaccination should be considered.

## Introduction

*Salmonella enterica* serovar Typhi causes an estimated 22 million cases of typhoid fever and 216,000 deaths annually worldwide [1]. Humans are the only known reservoir and infection is usually transmitted through contaminated food or water [2]. Systemic illness usually presents with fever, headache, and abdominal pain, however, multiple severe complications can occur, including intestinal hemorrhage, intestinal perforation, hepatitis, pneumonia, and neuropsychiatric abnormalities [3].

Access to piped, treated water, modern sanitation, and safer food production have nearly eliminated typhoid fever as a public health problem in developed countries [4]. However, in developing countries, where investments in water and sanitation infrastructure have not kept pace with growing needs, household measures to prevent transmission of enteric illness, including typhoid fever, are needed [5–6]. Recommended household prevention measures include treatment of household drinking water with point-of-use chlorination or filtration, safe water storage, discouragement of open defecation, construction of household latrines, and education on hygiene practices, including hand washing with soap and safe food handling [6–7]. Additional interventions, including the use of typhoid vaccines, have been considered to prevent typhoid transmission in the outbreak setting [8–9].

In sub-Saharan Africa, typhoid fever causes an estimated 233 cases per 100,000 persons per year; while non-typhoidal *Salmonella* is likely endemic in Malawi and Mozambique, the prevalence of typhoid fever in these countries is not well characterized [1,10–16]. In March–November 2009, an outbreak of unexplained febrile illness with neurologic complications was investigated along the remote Malawi-Mozambique border and determined to be caused by typhoid fever [17]. The number of reported cases of typhoid fever increased 340% from July to September 2010, by which time a total of 748 illnesses and 44 deaths had been reported [18]. Despite efforts to improve access to safe water and sanitation, including limited construction of borehole wells and latrines in the affected area, promotion and free distribution of point-of-use household water chlorination products and soap, and targeted educational campaigns focusing on household water treatment and safe storage, hand washing with soap, safe food preparation, and proper sanitation, cases of typhoid fever continued to be reported [18]. In September 2010, 18 months after the start of the typhoid fever outbreak, we assessed household knowledge, attitudes, and practices (KAP) to better understand the impact of previous typhoid fever prevention efforts and inform future efforts in the region.

## Materials and methods

### Study area and respondents

Neno District is in a remote and mountainous region in southwestern Malawi, bordering Mozambique. Villages within Neno District vary with respect to accessibility by road, distance to health centers, and existing water and sanitation infrastructure. All 17 villages in Neno District affected by the typhoid fever outbreak in 2009–2010 were included in the survey. These villages were within a 14-kilometer radius and have an estimated population of 18,139 (Neno District Health Officer, unpublished). By the start of the KAP survey in September 2010, interventions in 15 of the 17 affected villages included community meetings (“typhoid talks”) about causes and prevention of typhoid fever, the importance of household water treatment and safe storage, hand washing with soap, safe food preparation, and adequate sanitation, that were coupled with free distribution of soap and WaterGuard, a locally produced, dilute sodium hypochlorite solution for chlorination of household drinking water. To help promote behavior change, “typhoid talks” were developed and led by district health office and non-governmental organization staff. Key messages were reinforced using a locally produced DVD about the typhoid outbreak, dramatic performances, posters and flyers, and live demonstrations of water treatment using recommended treatment products, hand washing, and construction of hand washing stations. All community members were encouraged to attend, and time was allocated for answering questions posed by attendees. Six of 17 villages also received improved infrastructure, including construction of borehole wells and pit latrines (Table 1). An estimate of the number of households in each village was provided by village leadership. The female head of household was selected as the target respondent for the survey as she would be expected to be the most knowledgeable about household water, sanitation and hygiene practices.

**Table 1:** Interventions received by village, as reported by village leadership, and number and percentage of surveyed household respondents who reported attendance at community educational meetings (“typhoid talks”), September, 2010.

Village	Post-Outbreak Interventions in Villages			Enrolled Households		
	Community Meeting or "Typhoid Talk"	WaterGuard Distributed	Infrastructure Improvements*	Number Households Enrolled	Reported "Typhoid Talk" Attendance	
					n	(%)
**Chakulembera**	Yes	Yes	No	11	7	(63)
**Chikalema**	No	No	No	12	4	(33)
**Chimbalanga I**	Yes	Yes	Yes	12	4	(33)
**Chimbalanga II**	Yes	Yes	No	12	4	(33)
**Chiyembekeza**	Yes	Yes	No	12	9	(75)
**Kagudza**	Yes	Yes	No	12	7	(58)
**Kaingilira**	Yes	Yes	No	12	7	(58)
**Kalimedzako**	Yes	Yes	Yes	12	4	(33)
**Kamoto**	No	No	No	12	4	(33)
**Kumbwani**	Yes	Yes	Yes	12	4	(33)
**Kundembo**	Yes	Yes	Yes	12	5	(42)
**Kweneza**	Yes	Yes	No	12	11	(92)
**Masamba**	Yes	Yes	Yes	12	8	(67)
**Moffat**	Yes	Yes	No	12	8	(67)
**Mposadala**	Yes	Yes	No	12	8	(67)
**Mtemankhawa**	Yes	Yes	Yes	12	10	(83)
**Nseula**	Yes	Yes	No	11	7	(64)

*Infrastructure improvements include construction of improved water sources (boreholes and protected springs) and construction of pit latrines

### Questionnaire design

A household questionnaire (S1 Fig) was developed by the Centers for Disease Control and Prevention (CDC), the Neno District Health Office, and non-governmental partner organizations involved in post-outbreak response activities. It was designed to assess knowledge regarding the causes, treatment, and methods of preventing typhoid fever and household knowledge, attitudes and practices on water, sanitation, and hygiene. Household water treatment practices were reported for the 2 weeks before administration of the household questionnaire (i.e., in 2010) and during the previous year (i.e., in 2009). The final survey was administered by trained enumerators in the local language, Chichewa.

### Household surveys

In each of the 17 villages, households were randomly selected. Enumerators located the central point of each village and determined a random direction for household sampling by spinning a bottle. The first house encountered in which the female head of household was available and willing to participate was enrolled. If the female head of household was unavailable or unwilling to participate, an adjacent household was substituted. Subsequent households were selected using a pre-determined skip pattern; if the estimated number of households in the village was less than 150, then 3 households were skipped and if there were more than 150 households, then 6 households were skipped. A new direction of sampling was selected from the central starting point if the enumerator reached the edge of the village or the border with Mozambique. Twelve households were surveyed per village; 12 was chosen based upon available time and resources.

### Household stored drinking water testing

In all households in which drinking water was stored and available for testing at the time of the visit, samples were collected and tested for free chlorine residual (FCR) using the N,N diethyl-p-phenylene diamine (DPD) colorimetric method (HACH Company, Loveland, CO, USA). FCR levels were considered adequate at ≥ 0.2 milligrams per liter [19].

### Water source testing

Two water samples were collected from improved drinking water sources in 8 of 17 survey villages, including 9 boreholes and 3 taps. Water samples were tested for total coliform bacteria and *Escherichia coli* using presence-absence broth with 4-methylumbelliferyl-β-D-glucuronide (HACH method 8364, HACH Company, Loveland, CO, USA).

### Data management and analysis

Data were entered into a Microsoft Access 2007 database (Redmond, WA, USA) and analyzed using SAS version 9.3 (Cary, NC, USA). Attendance at a “typhoid talk” was not known in advance of the administered household survey and so, random samples could not be independently drawn from households that attended a “typhoid talk” and households that did not attend. However, response frequencies were computed for all household respondents and stratified by those who reported attending a “typhoid talk” and those who did not. To identify differences between these two groups, comparisons of proportions were conducted using the Rao-Scott design-adjusted chi-square test accounting for village clusters. Differences were evaluated for statistical significance at the alpha = 0.05 level.

### Ethics

This survey was initiated in the setting of an ongoing typhoid fever outbreak in an effort to guide additional interventions. Human subjects research designees at CDC and on the Malawi National Human Subjects Review Committee determined that this activity constituted public health response and program evaluation rather than research. Verbal permission to enroll households within a village and collect water from improved water sources was obtained from village leadership. Female heads of household provided verbal consent for their participation in the survey and testing of household stored water. All consents were obtained in Chichewa.

## Results

### Household survey

A total of 393 households were visited and 202 (51%) were enrolled in 17 villages; 187 (48%) female heads of household were unavailable and 4 (1%) refused participation. Among enrolled households (n = 202), the median age of respondents was 30 years (range 18–83 years) (Table 2). The median household size was 5 persons (range 1–12) and the median number of children under 5 years of age was 1 (range 0–4). Some formal education (i.e., any attendance in a primary or secondary school) was reported by 78% of respondents and 57% reported being able to read. Among the household assets included in the survey (bicycle, motorcycle, car, radio, television, refrigerator, solar panel), ownership of a radio was most common (69%); 26% of households reported owning none of the household assets.

**Table 2:** Demographic characteristics of households and respondents enrolled in knowledge, attitudes, and practices survey, Neno District, Malawi, September 2010.

	Total		Attended "Typhoid Talk"		Did not attend "Typhoid Talk"	
	(N = 202)		(N = 111)		(N = 87)	
	n	(%)	n	(%)	n	(%)
**Demographic Characteristics**						
Median age in years (range)	30	(18–83)	30	(18–83)	32	(18–82)
Median no. of people in household (range)	5	(1–12)	5	(1–11)	5	(2–12)
Median no. less than 5 years in household (range)	1	(0–4)	1	(0–3)	1	(0–4)
Self-reported literacy*	116	(57)	71	(64)	44	(51)
Any formal education*	158	(78)	92	(83)	64	(74)
No formal education*	44	(22)	19	(17)	23	(26)
Household Assets*						
None	52	(26)	28	(25)	23	(26)
Radio	139	(69)	77	(69)	59	(68)
Bicycle	81	(40)	46	(41)	34	(39)
Solar panel	36	(18)	21	(19)	15	(17)
Television	20	(10)	14	(13)	6	(7)
Motorcycle	6	(3)	3	(3)	3	(3)
Car	0	(0)	0	(0)	0	(0)
Refrigerator	0	(0)	0	(0)	0	(0)

*Self-reported literacy, any formal education, no formal education, and household assets were similar between the two groups using the P < 0.05 by Rao-Scott design-adjusted chi-square test accounting for clustering by village (significance considered at P < 0.05).

One hundred and eleven (56%) of 198 household respondents in 17 villages reported attending a “typhoid talk”; four respondents in 4 villages were unsure. Reported attendance ranged from 33% of respondents in six villages, including the two villages where no talks were given, to 92% in Kweneza. Formal education, self-reported literacy, and household assets were not significantly different among those who reported attending a “typhoid talk” compared with those that did not. Respondents who attended a “typhoid talk” (n = 111) reported that talks were led by community health workers (67%), clinicians (29%), and non-governmental organizations (14%). Of 111 respondents who attended a “typhoid talk”, 85 (77%) reported receiving free products at the talk. Of these, 86% received WaterGuard and 78% received soap.

Among all household respondents (n = 202), the most commonly reported causes of typhoid fever were poor hygiene (77%), drinking unsafe water (49%), and consuming unsafe food (25%) (Table 3). Boiling or treating drinking water (68%), hand washing (52%), and cleaning cooking utensils and vessels (38%) were the most commonly reported methods for preventing typhoid fever. Among those that reported attending a “typhoid talk” (n = 111), reported causes of typhoid fever were poor hygiene (86%), drinking unsafe water (54%), and consuming unsafe food (30%). Also, these respondents reported that boiling or treating drinking water (73%), hand washing (52%), and cleaning cooking utensils and vessels (45%) were methods for preventing typhoid fever. Cleaning cooking utensils and vessels was more commonly reported among respondents who attended a “typhoid talk” compared with those who did not (P = 0.0261). Most respondents (98%), regardless of their reported “typhoid talk” attendance (n = 202), indicated that they would seek treatment for typhoid fever at a hospital or clinic, while only 2% reported use of a home remedy.

**Table 3:** Knowledge of causes, prevention methods, and treatment of typhoid fever among survey respondents, Neno District, Malawi, September 2010.

	Total		Attended "Typhoid Talk"		Did not attend "Typhoid Talk"	
	(N = 202)		(N = 111)		(N = 87)	
	n	(%)	n	(%)	n	(%)
**Causes of typhoid fever**						
Poor hygiene*	155	(77)	96	(86)	59	(68)
Drinking unsafe water	98	(49)	60	(54)	38	(44)
Consuming unsafe food	50	(25)	33	(30)	17	(20)
Flies	15	(7)	11	(10)	4	(5)
Unwashed fruits and vegetables	12	(6)	6	(5)	6	(7)
Person-to-person spread	1	(1)	1	(1)	0	(0)
Omens	0	(0)	0	(0)	0	(0)
People from other tribes	0	(0)	0	(0)	0	(0)
Other	17	(8)	7	(6)	10	(11)
Don't Know*	25	(12)	6	(5)	15	(17)
**Methods of preventing typhoid fever**						
Cannot prevent	2	(1)	0	(0)	2	(2)
Boil or treat water	137	(68)	81	(73)	55	(63)
Wash hands	105	(52)	58	(52)	47	(54)
Clean cooking utensils and vessels*	76	(38)	50	(45)	24	(28)
Cook food thoroughly	68	(34)	36	(32)	32	(37)
Wash vegetables and fruits	35	(17)	15	(14)	20	(23)
Other	21	(10)	13	(12)	8	(9)
Don't Know*	10	(5)	1	(1)	7	(8)
**Treatment of typhoid fever**						
Do not treat	0	(0)	0	(0)	0	(0)
Go to clinic or hospital	197	(98)	109	(98)	85	(98)
Home remedy	4	(2)	2	(2)	2	(2)
Traditional healer	0	(0)	0	(0)	0	(0)
Other	3	(1)	3	(3)	0	(0)
Don't Know	2	(1)	0	(0)	1	(1)

*P < 0.05 by Rao-Scott design-adjusted chi-square test accounting for clustering by village: Causes of typhoid fever: poor hygiene (p = 0.0155), don’t know (p = 0.0330); methods of preventing typhoid fever: cleaning cooking utensils and vessels (p = 0.0261), don’t know (p = 0.0254).

Among all households (n = 202), the primary sources of household drinking water were unimproved wells (45%), boreholes (42%), rivers (7%) and taps (5%); respondents who reported using a borehole as their primary water source (n = 84) also reported drinking water from unimproved wells (54%) and rivers (6%). Nearly all respondents (198, 98%) reported having ever treated their drinking water; among these, only 30% reported always treating their drinking water (Table 4). Among households who reported ever treating their drinking water (n = 198), WaterGuard was the most popular treatment method (93%) in 2010; additional treatment methods included use of homemade chlorine solution (25%) and boiling (15%). Reported use of WaterGuard increased from 32% of households in 2009 to 93% of households in 2010, while reported use of boiling decreased from 23% to 15%. Use of homemade chlorine solution remained unchanged during 2009–2010. Reported barriers preventing regular treatment of household drinking water (n = 137) included lack of WaterGuard in the home (77%), being too busy (20%), lack of homemade chlorine solution in the home (17%), belief that their current water source is safe (11%), and affordability (8%); only 3% of respondents reported bad taste or smell associated with the use chlorine-based treatment products as a barrier for regularly treating their water.

**Table 4:** Household safe water, hygiene, and sanitation practices among survey respondents, Neno District, Malawi, September 2010.

	Total		Attended "Typhoid Talk"		Did not attend "Typhoid Talk"	
	(N = 202)		(N = 111)		(N = 87)	
	n	(%)	n	(%)	n	(%)
**Treatment of stored drinking water**						
Ever treated drinking water	198	(98)	110	(99)	84	(97)
Always treat drinking water (N = 198*)	59	(30)	34	(31)	24	(29)
Methods for treating drinking water (N = 198*)						
WaterGuard	185	(93)	107	(97)	75	(89)
Homemade chlorine solution	50	(25)	28	(25)	21	(25)
Boiling	29	(15)	16	(15)	13	(15)
PUR†	3	(2)	2	(2)	1	(1)
Certeza‡	1	(1)	0	(0)	1	(1)
Other	2	(1)	0	(0)	2	(2)
**WaterGuard in the home**						
Received free WaterGuard	180	(89)	104	(94)	73	(84)
Purchased WaterGuard	17	(8)	8	(7)	9	(10)
Insufficient WaterGuard supply in past month	78	(39)	39	(35)	36	(41)
WaterGuard observed in household	112	(56)	63	(57)	48	(56)
**Storage of household drinking water (N = 176§)**						
Types of stored water vessels						
Wide-mouthed vessels	132	(75)	75	(77)	56	(73)
Narrow-mouthed vessels	46	(26)	24	(25)	21	(27)
At least one uncovered stored water vessel	61	(35)	34	(35)	27	(35)
Accessing stored drinking water						
Scoop water from vessel	132	(75)	75	(77)	56	(73)
Pour from vessel	46	(26)	24	(25)	21	(27)
**Soap and washing hands**						
Observed soap in household	154	(77)	83	(76)	69	(79)
Use soap to wash hands (N = 154)¶	79	(51)	35	(42)	43	(62)
When should you hand wash						
After using toilet	185	(92)	103	(93)	79	(91)
Before eating¶	148	(73)	90	(81)	55	(63)
After cleaning child who has defecated	100	(50)	59	(53)	39	(45)
Before cooking	80	(40)	47	(42)	33	(38)
Other	72	(36)	40	(36)	29	(33)
Don't Know	0	(0)	0	(0)	0	(0)
**Observed types of household latrines**						
Private latrine	114	(56)	67	(60)	45	(52)
Shared latrine	76	(38)	39	(35)	35	(40)
Open defecation	15	(7)	6	(5)	9	(10)

*Restricted to households who reported ever treating their drinking water

†PUR Purifier of Water^TM^ (Procter & Gamble, Cincinnati, OH, USA) product contains ferric sulfate (a flocculant) and calcium hypochlorite (a disinfectant)

‡Certeza, is a dilute sodium-hypochlorite solution marketed and distributed in Mozambique

§Households with stored drinking water vessels available for observation at the time of the household visit

¶P < 0.05 by Rao-Scott design-adjusted chi-square test accounting for clustering by village: use soap to wash hands (p = 0.0070) and should wash hands before eating (p = 0.0077).

Overall (n = 202), 89% of household respondents reported that they received free WaterGuard (Table 4). Few respondents (8%) reported having ever purchased WaterGuard, even though 39% reported an insufficient household supply of WaterGuard in the past month. Fifty-six percent of households were observed to have a bottle of WaterGuard at the time of interview; among these (n = 112), 34% reported that the water stored in their home was treated (Table 5). In households with drinking water storage vessels available for observation (n = 176), water was stored in a combination of vessels with mouths wide enough to allow a hand to pass through the opening and touch the stored water (75% of households) and narrow-mouthed vessels (26%), and 35% of these households had at least one uncovered drinking water storage vessel (Table 4). Seventy-five percent reported using a cup or ladle to scoop water from observed storage vessels.

**Table 5:** Reported use of WaterGuard to treat stored drinking water and results of free chlorine residual testing among surveyed households with an observed bottle of WaterGuard.

	Total		Attended "Typhoid Talk"		Did not attend "Typhoid Talk"	
	(N = 112)		(N = 63)		(N = 48)	
	n	(%)	n	(%)	n	(%)
**Reported water treatment (if bottle present)**	38	(34)	25	(40)	13	(27)
**Results of chlorine testing**	**(N = 38)**		**(N = 25)**		**(N = 13)**	
Free chlorine residual						
Adequate (≥0.2 mg/L)	29	(76)	20	(80)	9	(69)
Positive, but inadequate (<0.2 mg/L)	3	(8)	1	(4)	2	(15)
Negative	6	(16)	4	(16)	2	(15)

Seventy-seven percent of all households (n = 202) were observed to have soap; among households with soap (n = 154), 51% reported using it to wash hands (Table 4). Use of soap for washing hands was more commonly reported by household respondents who did not attend a “typhoid talk” (62%) compared with those that did (42%) (P = 0.0070). Respondents (n = 202) reported that hand washing should be performed after using the toilet (92%), before eating (73%), after washing and cleaning babies (50%), and before cooking (40%). Washing hands before eating was more commonly reported by respondents who attended a “typhoid talk” (81%) compared with those that did not (63%) (P = 0.0077). Privately owned (56%) and shared pit latrines (38%) were the most common reported sites of defecation; 7% reported open defecation.

### Laboratory investigation

Free chlorine residual levels were adequate (FCR ≥ 0.2 mg/L) in 29 (76%) of the 38 households who reported treatment of stored water and had stored water available for testing and an observed bottle of WaterGuard (Table 5). Among the 38 households, FCR levels were adequate in 80% of 25 that reported attending a “typhoid talk”, and in 69% of 13 that reported not attending a “typhoid talk”. Tests for bacterial coliforms and *E*. *coli* were positive in samples from 5 of 9 boreholes and all 3 public taps tested in survey villages.

## Discussion

Sixteen months following the onset of a major typhoid fever outbreak in Neno District, Malawi and after targeted education and prevention interventions by Ministry of Health and partner organizations, household knowledge of the causes and methods of preventing typhoid fever, and adoption of safe water, sanitation, and hygiene practices at the household level, were suboptimal. Educational activities reportedly did not reach almost one-half of the target population. Even among household respondents who did attend a community-wide educational activity, knowledge of the relationship between drinking unsafe water, poor hygiene and typhoid fever was less than ideal. Despite the distribution of free WaterGuard and soap, few households adopted point-of-use water treatment and improved hygiene practices into their regular household routines. Qualitative research conducted at the same time as this investigation revealed persistent underlying skepticism about waterborne transmission of typhoid fever and the effectiveness of water, sanitation, and hygiene interventions to prevent further disease transmission [20]. Beliefs that the outbreak started and spread widely because of an ancestral curse, witchcraft, and ‘bad air’ in combination with an unusual illness associated with rapidly progressive disease and often fatal outcomes and failure by healthcare clinicians to diagnose and treat the illness likely contribute to this skepticism [20]. These results highlight the need for more effective interventions to improve household knowledge of typhoid fever transmission and prevention, and increase uptake and maintenance of preventive behaviors including regular water treatment and hand washing.

Most households reported getting their household drinking water from unimproved sources, and all stored water in their homes for extended periods. In this setting, household treatment and safe storage of drinking water are universally applicable, but were practiced by fewer than half of all households; therefore, most households remained at high risk for waterborne diseases, including typhoid fever. One possible explanation for the low rate of household drinking water treatment was the lack of regular access to household chlorination products, including WaterGuard; while many households reported having received free WaterGuard, many also reported an insufficient free supply to treat all of their water and few purchased water treatment products. Efforts to distribute free WaterGuard reached most households. However, free WaterGuard supplies were limited, and in most households, free distribution was not sufficient to promote sustained behavior change and regular treatment of all stored drinking water. Uptake in the use of WaterGuard or other household water treatment products may also be hampered by low familiarity with these products before the outbreak and by underlying beliefs regarding other modes of transmission [20–21]. Free chlorine residual levels were adequate in most households who reported using WaterGuard and had a bottle in the home at the time of the visit, suggesting adherence to recommended product use instructions.

Knowledge and adoption of recommended hand washing practices was also limited. Only half of households with soap reported using it to wash hands. Hand washing behaviors at key times, including hand washing after cleaning a child who has defecated and before preparing meals, were reported by less than 50% of respondents. Latrine use, either of a shared or privately owned latrine, was high; however, open defecation was still reported by some households.

Over the past several years, outbreaks of typhoid fever have been documented in other sub-Saharan African nations where access to safe water and sanitation facilities remains limited [21–27]. The emergence of antimicrobial resistance in these outbreaks complicates case management and outbreak control, highlighting the need for effective, practical interventions to reduce the risk of typhoid fever transmission [17,22–23,27]. Household stored drinking water treatment, hand washing with soap, and latrine use are all recommended to reduce transmission of enteric illnesses, including typhoid fever. However, encouraging adoption of these practices in every household is challenging and published research describing the behavioral factors that influence adoption and sustained use of these prevention interventions is scarce [28–29]. Several factors could have contributed to poor uptake of recommended prevention methods. Reported barriers included an insufficient supply of water treatment products for regular use and time needed to treat water; unlike in other areas affected by typhoid fever outbreaks, few reported bad taste associated with water treatment products [21]. Other studies have reported similar findings, but also hypothesize that the cost of the recommended products, reluctance to purchase products previously received for free, beliefs about the safety of current water sources and the underlying mechanism of disease transmission, and the lack of ongoing campaigns to promote adoption of recommended behaviors hamper the uptake of recommended prevention practices [20,29–32]. Qualitative research conducted in the study area, including focus group discussions and in-depth interviews, found that communities perceived typhoid fever to be dangerous and highly contagious, yet widely-held beliefs about typhoid transmission through curses, witchcraft, and ‘bad air’ were incompatible with prevention recommendations that focused on water treatment, hygiene and sanitation [20]. Future efforts to change household behaviors will require addressing community concepts about typhoid fever disease causation and transmission and improving routine access to recommended household water treatment products and soap through creative, cost-effective approaches that leverage existing commercial channels [33]. Behavior change is a complex process that is more effective when it is based upon tested and accepted theories that enhance behavioral change and includes repeated interventions to increase knowledge and promote new practices [28]. In this case, efforts to improve safe water, hygiene, and sanitation practices in the affected population could be enhanced through repeated promotional efforts to increase familiarity with available products by community leaders and peers rather than through “one-off” village-level meetings and product distributions led by “outsiders” [34–36]. Furthermore, prevention efforts that occurred in neighboring affected villages in Mozambique, where water treatment products are marketed under a different name, were reported by the community as less intense. Better coordination by health agencies in both countries might have improved adoption of recommended prevention practices by all.

Limitations of this study include a high rate of target household respondent unavailability, the lack of a pre-intervention baseline evaluation for comparison, ongoing prevention activities during the evaluation period, and limited surveillance for typhoid fever. Respondents who were not available for enrollment because of work in the fields or travel outside the village may have responded differently to survey questions than those who were available for participation. The lack of a pre-intervention survey or prior knowledge of whether a household benefited from post-outbreak interventions made it impossible to randomly sample households from populations that either attended or did not attend these talks and to measure the impact that intervention efforts had on changing knowledge or altering practices within the home. Therefore, interpretation of tests of association should be done with caution. Ongoing prevention activities during the evaluation, including the distribution of WaterGuard, may have influenced household responses. Limited surveillance data from Neno District makes it difficult to associate the impact interventions may have had on knowledge, attitudes and practices in the home with the apparent decrease in typhoid fever illnesses and deaths.

In summary, despite ongoing outbreak interventions, including community-wide educational campaigns and distribution of WaterGuard and soap, knowledge regarding the causes and prevention of typhoid fever and ownership and use of products that help reduce disease transmission remained low, even among household respondents who reported benefiting from these interventions. Future efforts to improve household water quality and sanitation and hygiene practices need to be more forceful and sustained until deficiencies in improved water supply and sanitation infrastructure can be fully addressed. In the interim, the option to complement these efforts with a targeted typhoid fever vaccination campaign should be strongly considered [20].

## Supporting information

S1 Fig Malawi typhoid fever investigation: Community assessment survey. English-Chichewa community water, sanitation, and hygiene assessment survey conducted in Neno, Malawi, September, 2010.
(DOCX)
