# Supplementary material for: Assessment of water, sanitation and hygiene interventions in response to an outbreak of typhoid fever in Neno District, Malawi
Source: PLoS One. 2018 Feb 23;13(2):e0193348. doi: 10.1371/journal.pone.0193348 (PMC5825105; doi:10.1371/journal.pone.0193348)
Supplement: S1 Fig — English-Chichewa community water, sanitation, and hygiene assessment survey conducted in Neno, Malawi, September, 2010. (DOCX) [file pone.0193348.s001.docx]

**Questions:**

| **Q#** | **Question** | **Answer** | |
| --- | --- | --- | --- |
| **RESPONDENT DEMOGRAPHICS** | | | |
| **1.** | **Muli ndi zaka zingati?**  Respondent age in years? | | Years |
| **2.** | **Kodi mnyumba muno mumakhalamo anthu angati amene mamadyela nawo pamodzi?**  How many people live in your household, that is, share food from the same cooking pot? | | Total people |
| **3.** | **Nanga ana osachepera zaka zisanu alipo angati?**  How many children less than 5 years old? | | Children |
| **KNOWLEDGE OF TYPHOID** | | | |
| **4.** | **Ngati eya Kodi chimayambitsa typhoid ndi chiyani?**  *Do not read. Mark all that apply.*  What causes typhoid? | ❒**Kumwa madzi oyipa** (Drinking bad water)  ❒**Kudya chakudya chomwe sichaukhondo** (Eating bad food)  ❒**Kudya zipatso kapena masamba osatsuka** (Unwashed fruit/  vegetables)  ❒**Ntchentche / Tizilombo tina tating’ono ting’ono (**Flies/insects)  ❒**Kusowa ukhondo** (Poor hygiene)  ❒ **Mizimu / themberero / mafano** (Spirits/curse/bad omen)  ❒ **Anthu amakhala ku mudzi ina** (People from other tribes/villages)  ❒ **Munthu wa munthu** (Person-to-person)  ❒**China, tchulani** (Other, Specify) *_____________*___________  ❒**Sindikudziwa** (Don’t know)  ❒**Akana** (Refused) | |
| **5.** | **Kodi mungapewe bwanji typhoid kuti wapabanja panu asadwale?**  *Do not read. Mark all that apply.*  How can you prevent you or your family members from getting typhoid? | ❒**Sitetezeka** (Cannot prevent)  ❒**Kusamba m’manja tikachoka ku chumbudzi, tikasintha mwana**  **thewera, tisanadye, tisanakonze chakudya** (Wash hands)  ❒**Kuphika madzi** (Boil or treat water)  ❒**Kuona kuti chakudya chapsya moyenera** (Cook food thoroughly)  ❒**Kutsuka zipatso ndi ndiwo zamasamba tisanazidye kaye (**Wash  vegetables and fruit)  ❒**Kugwirirtsa ntchito ziwiya zotsukidwa bwino** (Clean cooking  utensils/ vessels)  ❒**Ngati pali zina tchulani** (Other)*_______ ___*  ❒**Sindikudziwa** (Don’t know)  ❒**Akana** (Refused) | |
| **6.** | **Inuyo kapena pap banja panu munagaichize bwanji typhoid?**  *Do not read. Mark all that apply.*  How would you treat typhoid for yourself or your family members? | ❒ **Imatha yokha (**Do not treat)  ❒ **Njira zina za pakhomo** (Home remedy)  ❒ **Kwa a sing’anga** (Go to a traditional/herbal healer)  ❒ **Kuchipatala** (Go to clinic/hospital)  ❒ **Ngati pali njira zina tchulani** (Other, specify)*__________*  ❒ **Sindikudziwa (**Don’t know)  ❒ **Akana (**Refused) | |
| **7.** | **Kuchokera chaka chino, alipo wina amene anakuphunzitsani za kapewedwe ka matenda a typhoid?**  Since the beginning of this year, has anyone talked to you about preventing and treating typhoid? | ❒**Eya** (Yes)  ❒**Ayi (**No) **→ GO TO 11**  ❒**Sindikudziwa (**Don’t know) **→ GO TO 11**  ❒**Akana (**Refused) **→ GO TO 11** | |
| **8.** | **Ndi ndani anakuuzani za typhoid?**  *Do not read. Mark all that apply.*  Who talked to you about typhoid? | ❒ **Wachibale** (Family member)  ❒ **Oyandikana nawo nyumba** (Neighbor)  ❒ **Nzanu (**Friend)  ❒ **Wazaumoyo (**Community health worker/HSA)  ❒ **Adokotala**, **a ku** (Clinician, specify clinic)___________________  ❒ **Wailesi** (Radio)  ❒ **Munyuzi** (Newspaper)  ❒**Pa pepala** (Brochure/flyer)  ❒ **Pansonkhano (**Community meeting with health workers (CHW,  HSA, Clinician))  ❒ **Pansonkhano** (Community meeting with PSI/UNICEF)  ❒ **Masewelo** (Actors in drama group performances)  ❒**Mafilimu** (Movie / DVD / video)  ❒ **Ngati ndi kwina kwake tchulani** (Other, specify)_____________  ❒**Sindikudziwa (**Don’t know)  ❒**Akana (**Refused) | |
| **9.** | **Munalandila china chake kumene amakuuzani za matendawa?**  Were you given any product or materials when they talked to you about typhoid? | ❒**Eya** (Yes)  ❒**Ayi (**No) **→ GO TO 11**  ❒**Sindikudziwa (**Don’t know) **→ GO TO 11**  ❒**Akana (**Refused) **→ GO TO 11** | |
| **10.** | **Ngati eya, anakupatsani chani?**  *Read answers. Mark all that apply.*  If yes, what were you given? | ❒ Waterguard  ❒ Certeza  ❒ PuR  ❒ **Kololini** (Chlorine solution (1% stock solution))  ❒ **Sopo** (Soap)  ❒ **Mauthenga a papepala** (Print Materials)  ❒ **Malangizo** (Advice or information)  ❒ **T-shirt**  ❒ **Ngati pali china tchulani** (Other, specify)____________________  ❒**Sindikudziwa (**Don’t know)  ❒**Akana (**Refused) | |
| **DRINKING WATER SOURCE** | | | |
| **11.** | **Kodi mumatunga kuti madzi anu okumwa?**  *Do not read. Mark only one answer. If they say multiple answers, ask which was the primary source.*  What is the current source of drinking water for your household? | ❒ **Pa mpope** (Tap water)  ❒ **Mtsinje** (River)  ❒ **Dilawo** (Bore Hole) *Specify village:_________________________*  ❒ **Ku khwawa** (Stream)  ❒ **A m’botolo (**Bottled Water)  ❒ **Nyanja** (Lake)  ❒ **Chitsime** (Well)  ❒ **Ngati pali kwina atchule** (Other, specify)__________________  ❒**Sindikudziwa (**Don’t know)  ❒**Akana (**Refused) | |
| **12.** | **Kodi mumatunga kuti madzi anu okumwa nthawi ya chilimwe?**  *Do not read. Mark only one answer. If they say multiple answers, ask which was the primary source.*  What is the major source of drinking water for your household during the DRY season? | ❒ **Pa mpope** (Tap water)  ❒ **Mtsinje** (River)  ❒ **Dilawo** (Bore Hole) *Specify village: ________________________*  ❒ **Ku khwawa** (Stream)  ❒ **A m’botolo (**Bottled Water)  ❒ **Nyanja** (Lake)  ❒ **Chitsime** (Well)  ❒ **Ngati pali kwina atchule** (Other, specify)__________________  ❒**Sindikudziwa (**Don’t know)  ❒**Akana (**Refused) | |
| **13.** | **Kodi mumatunga kuti madzi anu okumwa nthawi ya dzinja?**  *Do not read. Mark only one answer. If they say multiple answers, ask which was the primary source.*  What is the major source of drinking water for your household during the RAINY season? | ❒ **Pa mpope** (Tap water)  ❒ **Mtsinje** (River)  ❒ **Dilawo** (Bore Hole) *Specify village: ________________________*  ❒ **Ku khwawa** (Stream)  ❒ **A m’botolo (**Bottled Water)  ❒ **Nyanja** (Lake)  ❒ **Chitsime** (Well)  ❒ **Ngati pali kwina atchule** (Other, specify)__________________  ❒**Sindikudziwa (**Don’t know)  ❒**Akana (**Refused) | |
| **14.** | **Pali kwinanso kumene munatungako madzi kupatula kumene mwatchulaku?**  *Read answers. Mark all that apply.*  From what other sources do you get your drinking water besides the ones mentioned above? | ❒ **Pa mpope** (Tap water)  ❒ **Mtsinje** (River)  ❒ **Dilawo** (Bore Hole) *Specify village: ________________________*  ❒ **Ku khwawa** (Stream)  ❒ **A m’botolo (**Bottled Water)  ❒ **Nyanja** (Lake)  ❒ **Chitsime** (Well)  ❒ **Ngati pali kwina atchule** (Other, specify)__________________  ❒**Sindikudziwa (**Don’t know)  ❒**Akana (**Refused) | |
| **15.** | **Kodi munasintha kumene mumatunga madzi anu okumwa chaka chino?**  Have you changed where you get your drinking water this year? | ❒**Eya** (Yes)  ❒**Ayi (**No) **→ GO TO 17**  ❒**Sindikudziwa (**Don’t know) **→ GO TO 17**  ❒**Akana (**Refused) **→ GO TO 17** | |
| **16.** | **Kodi mumatunga kuti madzi anu okumwa chaka chata?**  *Do not read. Mark only one answer. If they say multiple answers, ask which was the primary source.*  What was the major source of your drinking water last year? | ❒ **Pa mpope** (Tap water)  ❒ **Mtsinje** (River)  ❒ **Dilawo** (Bore Hole) *Specify village:*________________________  ❒ **Ku khwawa** (Stream)  ❒ **A m’botolo (**Bottled Water)  ❒ **Nyanja** (Lake)  ❒ **Chitsime** (Well)  ❒ **Ngati pali kwina atchule** (Other, specify)__________________  ❒**Sindikudziwa (**Don’t know)  ❒**Akana (**Refused) | |
| **17.** | **M’mudzi muno mwakumbidwa mjigo chaka chino?**  Was a new borehole or protected spring added in your village this year? | ❒**Eya** (Yes)  ❒**Ayi** (No) **→ GO TO 20**  ❒**Sindikudziwa** (Don’t know) **→ GO TO 20**  ❒**Akana (**Refused) **→ GO TO 20** | |
| **18.** | **Mumakatunga madzi ku mjigo watsopanowo?**  Do you get your drinking water from the new borehole well? | ❒ **Sindinukatunyepo** (Never)  ❒ **Nthawi zina** (Sometimes) **→ GO TO 20**  ❒ Nt**hawi zonse** (Always) **→ GO TO 20**  ❒**Sindikudziwa (**Don’t know) **→ GO TO 20**  ❒**Akana (**Refused) **→ GO TO 20** | |
| **19.** | **Ngati simunakatungeko ndichifukwa chiyani?**  *Do not read. Mark all that apply.*  If never, why not? | ❒ **Ndikutali kwambiri** (Too far away)  ❒ **Madziwo sakusangalatsani makomedwe ake** (Don’t like taste of  water)  ❒ **Kumakhala anthu ambiri** (Too many other people use it)  ❒ **China, tchulani (**Other, specify)___________________________  ❒**Sindikudziwa (**Don’t know)  ❒**Akana (**Refused) | |
| **20.** | **Kodi mumalipira ndalama zingati madziwo?**  How much do you pay for your water? | ❒**Palibe** (Nothing)  ____________ **Pa chiwiya chimodzi** (Per container)  ____________ **Pa (tchulani)** (Per (specify))___________________  ❒ **Sindikudziwa** (Don’t know)  ❒ **Akana** (Refused) | |
| **HOME STORED DRINKING WATER** | | | |
| **21.** | **Kodi ndingaoneko komwe mumasungirako madzi anu okumwa?**  May I please see where you store your drinking water? | ❒**Eya** (Yes)  ❒**Kulibe (**Not available) **→ GO TO 25**  ❒**Ayi** **/ Akana (**No / Refused) **→ GO TO 25** | |
| **22.** | **Kuchokera mukuyang’ana kodi madziwo:**  *If more than one, mark all that apply.*  From observation, container(s) is: | ❒ **Avindikiridwa** (Covered)  ❒ **Ndiwosavindikiridwa** (Uncovered)  ❒ **Ali mchigubu** (Jerry can) | |
| **23.** | **Kuchokera mukuyang’ana kukamwa kwa chosungira:**  *If more than one, mark all that apply.*  From observation, container opening is: | ❒ **Kukamwakwake ndikwakukulu koti dzanja likhonza kulowa**  (wide-mouthed (*hand can fit in the opening*))  ❒ **Kukamwa kwake mkwakung’ono ndipo dzanja silingalowe**  **(**narrow-mouthed (*too small for hand to fit through)*) | |
| **22.** | **Kodi chiwiyacho chili ndi mpope?**  *If more than one, mark all that apply.*  Does the container have a spigot top? | ❒**Eya** (Yes)  ❒**Ayi (**No) | |
| **25.** | **Mumachiti chani mukamaika madzi ena mu mtsuko wa madzi?**  *Do not read. Mark all that apply.*  What do you do when you renew the water in the storage container? | ❒**Kudzadzitsira madzi otsala** (Top off existing water in container)  ❒**Kuthiramo ena musanasuke chiwiyacho (**Refill when empty  without cleaning)  ❒**Kutsuka chiwiyacho ndikuthiramo madzi** (Clean container and  refill)  ❒**China, tchulani (**Other, specify)____________________________  ❒**Sindikudziwa (**Don’t know)  ❒**Akana (**Refused) | |
| **26.** | **Kodi madzi akumwawo mumachita kupungulira mu kapu kapena mumagwiritsa kapuyo potungira?**  *Mark all that apply.*  Is drinking water poured into a glass or cup, or does a person have to use a glass or cup to scoop it out? | ❒ **Madzi amathiridwa (**Water is poured out)  ❒ **Madzi amatungidwa** (Water is scooped out)  ❒ **China, tchulani** (Other, specify)___________________________  ❒**Sindikudziwa (**Don’t know)  ❒**Akana (**Refused) | |
| **27.** | **Mumagwiritsiranso ntchito zina kupatula kumwa?**  Do you use the water in this container for uses other than drinking? | ❒**Eya** (Yes)  ❒**Ayi (**No) **→ GO TO 29**  ❒**Sindikudziwa (**Don’t know) **→ GO TO 29**  ❒**Akana (**Refused) **→ GO TO 29** | |
| **28.** | **Mumagwiritsira nchito zake ziti?**  *Read answers. Mark all that apply.*  For what other uses? | ❒ **Kuphikira** (Cooking)  ❒ **Kutsukira ndiwo za masamba** (Washing vegetables)  ❒ **Kutsukira mbale ndi ziwiya zina** (Cleaning plates and utensils)  ❒ **Kuchapira zovala** (Washing clothes)  ❒ **Kusamba** (Bathing)  ❒ **China, tchulani (**Other, specify)___________________________  ❒**Sindikudziwa (**Don’t know)  ❒**Akana (**Refused) | |
| **IN HOME WATER TREATMENT** | | | |
| **29.** | **Munatetezako madzi anu akumwa powilista kapena munjira ina iliyonse?**  Have you ever treated your drinking water by boiling or by any other method? | ❒**Eya** (Yes) **→ GO TO 31**  ❒**Ayi** (No)  ❒**Sindikudziwa (**Don’t know) **→ GO TO 40**  ❒**Akana (**Refused) **→ GO TO 40** | |
| **30.** | **‘Madziwo simawateteza chifukwachani?**  *Do not read. Mark all that apply.*  If you do not treat your water at all, why not? | ❒ **Komwe ndimatunga madzi, nkotetezeka madziwo safunikanso**  **kuwateteza munjila ina** (My current water source is safe/does not  need treatment) **→ GO TO 40**  ❒ **Sindinga kwanitse** (Can’t afford) **→ GO TO 40**  ❒ **Tilibe mankhwala a chlorine** (No chlorine in the house) **→ GO**  **TO 40**  ❒ **Tilibe Watergard ndi Certeza ku nyumba** (No Waterguard or  Certeza in the home) **→ GO TO 40**  ❒ **Ndine otanganidwa** (Too busy) **→ GO TO 40**  ❒ **Simusangalatsidwa ndifungo lchlorine (**Children/Spouse  complained about the taste/smell) **→ GO TO 40**  ❒ **Ngati pali chifukwa china tchulani** (Other, specify)___________  __________________________ **→ GO TO 40**  ❒**Sindikudziwa (**Don’t know) **→ GO TO 40**  ❒**Akana (**Refused) **→ GO TO 40** | |
| **31.** | **Mumawateteza madzi anu okumwa chifukwa chiyani?**  *Do not read. Mark all that apply.*  Why do you treat your drinking water? | ❒ **Amakulu otetezeka** (Believe it makes it safer) ❒ **Tinalundila chlorine waulele** (Received water treatment  (Waterguard, Certeza) for free)  ❒ **Anzanu kapena achibale anaku uzani kuti ndi kofunika kutero**  (Your family or friends told you it was important)  ❒ **Kroywila ntchito zuchiputala anuku uzuni kuti ndikofunika**  (Someone at health facility told you it was important)  ❒ **Wazaumoyo anabwela mudzi mwanu kuzakuuzani njila ndi**  **kufunika ko teteza madzi** (A health worker visited your village to  talk to you about it)  ❒ **Amamyeka bwino** (Makes the taste better)  ❒ **Ngati pali china tchulani** (Other, specify)____________________  ❒**Sindikudziwa (**Don’t know)  ❒**Akana (**Refused) | |
| **32.** | **Kodi madzi anu akumwa mumawateteza powaphitsa kapena pali njira zina?**  *Refer to previous 2 weeks.*  Do you currently treat your drinking water by boiling or by any other method? | ❒**Eya** (Yes) **→ GO TO 34**  ❒**Ayi** (No)  ❒**Sindikudziwa (**Don’t know) **→ GO TO 34**  ❒**Akana (**Refused) **→ GO TO 34** | |
| **33.** | **‘Madziwo simuwateteza chifukwachani?**  *Refer to previous 2 weeks. Do not read. Mark all that apply.*  If you do not currently treat your water, why not? | ❒ **Komwe ndimatunga madzi, nkotetezeka madziwo safunikanso**  **kuwatoteza munjila ina** (My current water source is safe/does not  need treatment)  ❒ **Sindinga kwanitse** (Can’t afford)  ❒ **Tilibe mankhwala a chlorine** (No chlorine in the house)  ❒ **Tilibe Watergard ndi Certeza ku nyumba** (No Waterguard or  Certeza in the home) ❒ **Ndine otanganidwa** (Too busy)  ❒ **Simusangalatsidwa ndifungo chlorine (**Children/Spouse  complained about the taste/smell)  ❒ **Ngati pali chifukwa china tchulani** (Other, specify) ___________  **________________________**  ❒**Sindikudziwa (**Don’t know)  ❒**Akana (**Refused) | |
| **34.** | **Chaka chino, kodi mumagwiritsa ntchito chiyani poteteza madzi anu?**  *Read answers. Mark all that apply.*  This year, what have you used to treat your water? | ❒ **Kuphitsa** (Boiling)  ❒ Waterguard  ❒ Certeza  ❒ PuR  ❒ **Kololini** (Chlorine solution)  ❒ **Ngati pali kwina atchule** (Other, specify) ___________________  ❒**Sindikudziwa (**Don’t know)  ❒**Akana (**Refused) | |
| **35.** | **Kuyambira mu chaka chino kufika pano,** **kodi madzi anu mumawateteza kangati musanamwe?**  *Mark only one.*  From the start of this year to now, how often do you treat your drinking water before using it? | ❒ **Nthawi zonse** (Always) **→ GO TO 37**  ❒ N**thawi zina** (Some of the time)  ❒**Sindikudziwa (**Don’t know)  ❒**Akana (**Refused) | |
| **36.** | **Kuyambila chaka chino chifukwa chani nthawi zina simumateteza madzi anu ?**  *Read answers. Mark all that apply.*  Since the start of this year, why do you sometimes not treat your water? | ❒ **Ndikuganiza kuti madziwo ndiwotetezeka** (When I think the  source is safe)  ❒ **Ayi ndalama/sindinga kwanitse** (No money/cannot afford)  ❒ **Kolorini osungunula Mnyumba mulibe** (No chlorine solution in  the house)  ❒ **Tilibe Watergard ndi Certeza ku nyumba** (No Waterguard or  Certeza in the home)  ❒ **Ndine otanganidwa** (Too busy)  ❒ **Ana/Mabanja akudandaula za kakomedwe/fungo kakololini**  (Children/Spouse complained about the taste/smell)  ❒ **Ngati pali zina afotokoze** (Other, specify)___________________  ❒**Sindikudziwa (**Don’t know)  ❒**Akana (**Refused) | |
| **37.** | **Chaka chata, kodi madzi anu akumwa mumawateteza powaphitsa kapena pali njira zina?**  Last year, did you treat your drinking water by boiling or by any other method? | ❒ **Eya** (Yes)  ❒ **Ayi** (No) **→ GO TO 40**  ❒**Sindikudziwa (**Don’t know) **→ GO TO 40**  ❒**Akana (**Refused) **→ GO TO 40** | |
| **38.** | **Kodi chaka chatha, mumagwiritsa ntchito chiyani poteteza madzi anu?**  *Read answers. Mark all that apply.*  Last year, what did you use to treat your water? | ❒ **Kuphitsa** (Boiling)  ❒ Waterguard  ❒ Certeza  ❒ PuR  ❒ **Kololini** (Chlorine solution)  ❒ **Ngati pali kwina atchule** (Other, specify) ___________________  ❒**Sindikudziwa (**Don’t know)  ❒**Akana (**Refused) | |
| **39.** | **Chaka chata kufika pano,** **kodi madzi anu mumawateteza kangati musanamwe?**  *Mark only one.*  Last year, how often did you treat your drinking water before using it? | ❒ **Nthawi zonse** (Always)  ❒ N**thawi zina** (Some of the time)  ❒**Sindikudziwa (**Don’t know)  ❒**Akana (**Refused) | |
| **WATERGUARD** | | | |
| **40.** | **Chaka chino, mwalandilap chlorine kapena certzan waulele?**  Have you received free Waterguard or Certeza this year? | ❒**Eya** (Yes)  ❒**Ayi** (No) **→ GO TO 44**  ❒**Sindikudziwa (**Don’t know) **→ GO TO 44**  ❒ **Akana (**Refused) **→ GO TO 44** | |
| **41.** | **Mwalandilapo kangati waterguard kapena Certeza waulele chaka chino?**  *Mark only one.*  How many times have you received free Waterguard or Certeza this year? | ❒ **Kamodzi** (Once)  ❒ **Kupitilika kamodzi** (More than once) *Specify number of times:___*  ❒**Sindikudziwa (**Don’t know)  ❒**Akana (**Refused) | |
| **42.** | **Mwalandilapo mabotolo angati awaterguard aulele chaka chino?**  *Mark only one.*  How many free bottles of Waterguard have your received this year? | ❒ **Limodzi kapena awiri (**1-2 bottles)  ❒ **Mabotolo atatu kapena anai (**3-4 bottles)  ❒ **Mabotolo asanu (**5 bottles)  ❒ **Mabotolo ambiri, angati (**more than 5 bottles) *Specify number*  *bottles:______*  ❒**Sindikudziwa (**Don’t know)  ❒**Akana (**Refused) | |
| **43.** | **Ndiliti lomaliza limene munalandila waterguard waulele?**  *Mark only one.*  When was the last time you received free Waterguard? | ❒ **Mulungu uno (**Within the last week)  ❒ **Mwezi uno (**Within the last month)  ❒ **Kupitilia mwezi watha (**More than one month ago)  *Specify:__________________*  ❒**Sindikudziwa (**Don’t know)  ❒**Akana (**Refused) | |
| **44.** | **Mwagulapo kangati waterguard kapena certezachaka chino?**  *Mark only one.*  How many times have you bought Waterguard or Certeza this year? | ❒ **Sindimagulapo Watergard (**Never) **→ GO TO 46**  ❒ **Imodzi-iwiri kapena (**1-2 times)  ❒ **Itatu-inai kapena (**3-4 times)  ❒ **Isanu (**5 times)  ❒ **Kupitilila kasnu (**more than 5 times) *Specify number times:______*  ❒**Sindikudziwa (**Don’t know)  ❒**Akana (**Refused) | |
| **45.** | **Kodi munagula ndalama zingati ku Watergard kapena Certeza?**  How much did you pay for this Waterguard or Certeza? | ____________ **Pa chiwiya chimodzi** (Per container)    ❒**Sindikudziwa (**Don’t know)  ❒**Akana (**Refused) | |
| **46.** | **Kodi mwezi watha munali ndi waterguard wokwanila woteteza madzi anu nthawi zonse?**  In the last month, have you had sufficient Waterguard supply to treat all of your water every time ? | ❒**Eya** (Yes)  ❒**Ayi** (No)  ❒**Sindikudziwa (**Don’t know)  ❒ **Akana (**Refused) | |
| **47.** | **Mumadziwa bwanji kuti madzi anu okumwa ndi abwino, kapena oipa?**  *Do not read. Mark all that apply.*  How do you know that the safety of the drinking water is good or poor? | ❒ **Fungo lake** (By the smell)  ❒ **Kukoma kwake** (Taste)  ❒ **Maonekedwe ake** (Appearance)  ❒ **Anthu amadwala** (People get sick)  ❒ **Ana amadwala** (Children get sick)  ❒ **Palibe yemwe amadwala** (No one gets sick)  ❒ **Ndiwotetezedwa** (It is protected)  ❒ **Ziwiya zathu zimakhala zaukhondo ndi zovindukira**  (Containers are clean and covered)  ❒**Sindikudziwa (**Don’t know)  ❒**Akana (**Refused) | |
| **48.** | **Kodi ndi matenda anji amene angadze chifukwa chokumwa madzi osatetezedwa?**  *Do not read. Mark all that apply.*  Which diseases can result from drinking unsafe water? | ❒ Typhoid  ❒ **Kutsegula mmimba** (Diarrhea)  ❒ **Kolera** (Cholera)  ❒ **Kutsegula mmimba mwakamwazi** (Dysentery)  ❒ **Kutentha thupi** (Fever)  ❒ **Ngati pali zina afotokoze** (Other, specify)____________________  ❒**Sindikudziwa (**Don’t know)  ❒**Akana (**Refused) | |
| **49.** | **Kodi madzi ooneka bwino angakudwalitseni?**  Can water that looks clear make you sick? | ❒**Eya** (Yes)  ❒**Ayi** (No)  ❒**Sindikudziwa (**Don’t know)  ❒**Akana (**Refused) | |
| **50.** | **Kodi madzi amene mukumwa pa nyumba pano ndi ofunika kutetezedwanso mujanjila ina?**  Does the protection of the drinking water in your house need to be improved? | ❒**Eya** (Yes)  ❒**Ayi** (No)  ❒**Sindikudziwa (**Don’t know)  ❒**Akana (**Refused) | |
| **HYGIENE** | | | |
| **51.** | **Ndinthawi iti imene muyenela kusamba m’manja?**  *Do not read. Mark all that apply.*  When should you wash your hands? | ❒ **Ndikachoka ku chumbudzi** (After using the toilet)  ❒ **Ndisanadye** (Before eating)  ❒ **Ndikamaliza kudya** (After eating)  ❒ **Ndisanayambe kukonza chakudya** (Before cooking)  ❒ **Tikamaliza kusintha mwana thewera** (After washing/cleaning  babies)  ❒ **Ngati pali zina afotokoze** (Other, specify)____________________  ❒ **Sindisamba m’manja** (Never wash hands)  ❒**Sindikudziwa (**Don’t know)  ❒**Akana (**Refused) | |
| **52.** | **Kodi muli ndi sopo panyumba pano?**  Do you have soap in the house? | ❒ **Eya** (Yes)  ❒ **Ayi** (No) **→ GO TO 56**  ❒**Sindikudziwa (**Don’t know) **→ GO TO 56**  ❒**Akana (**Refused) **→ GO TO 56** | |
| **53.** | **Ndingawone nawo sopoyo?**  May I see the soap? | ❒ **Alipo** (Soap present)  ❒ **Palibe** (No soap in home) **→ GO TO 56**  ❒ **Akana** (Refused) **→ GO TO 56** | |
| **54.** | **Kodi sopoyo mumagwiritsa ntchito munjira zanji?**  *Do not read. Mark all that apply.*  For which purposes do you use the soap? | ❒ **Kusambira m’manja** (Washing hands)  ❒ **Kuchapira zovala** (Washing clothes)  ❒ **Kutsukira ziwiya** (Cleaning utensils/vessels)  ❒ **Kusambira** (Bathing)  ❒ **Ngati pali zina afotokoze** (Other, specify)____________________  ❒**Sindikudziwa (**Don’t know)  ❒**Akana (**Refused) | |
| **55.** | **Kodi pali malo amene mumasambira m’manja mukachoka ku chimbudzi?**  *Observe hand washing station. Must have water and soap in same location.*  Is there a place for washing hands after you leave the toilet? | ❒ **Eya** (Yes)  ❒ **Ayi** (No)  ❒ **Onani** (Unable to observe) *Reason:_________________________*  ❒**Akana (**Refused) | |
| **56.** | **Ndikuti kumene amthu pabanja lanu amapita kukazithandiza masana?**  *Read answers. Mark all that apply.*  Where do members of your household use the toilet during the day? | ❒ **Mokomo angapo chimbuzi chimodzi** (Shared latrine) *Specify*  *number households sharing: _______*  ❒ **Khomo limodzi chimbuzi chawo** (Own latrine)  ❒ **Kutchire** (In the bushes)  ❒ **Kupita paseri panyumba** (Next to house)  ❒ **Ngati pali zina afotokoze** (Other, specify)____________________  ❒**Sindikudziwa (**Don’t know)  ❒**Akana (**Refused) | |
| **57.** | **Ndikuti kumene pabanja lanu amapita kukazithandiza panthawi ya usiku?**  *Read answers. Mark all that apply.*  Where do members of your household use the toilet at night? | ❒ **Mokomo angapo chimbuzi chimodzi** (Shared latrine) *Specify*  *number households sharing: _______*  ❒ **Khomo limodzi chimbuzi chawo** (Own latrine)  ❒ **Kutchire** (In the bushes)  ❒ **Kupita paseri panyumba** (Next to house)  ❒ **Ngati pali zina afotokoze** (Other, specify)____________________  ❒**Sindikudziwa (**Don’t know)  ❒**Akana (**Refused) | |
| **58.** | **Kodi panyumba pano muli ndi chimbudzi?**  *Observe toilet / latrine.*  Is there a toilet or a pit latrine in the home? | ❒ **Eya** (Yes)  ❒ **Ayi** (No) **→ GO TO 60**  ❒ **Onani** (Unable to observe) *Reason:_________________________*  ❒**Akana (**Refused) **→ GO TO 60** | |
| **59.** | **Kodi chimbudzi chimenechi chinamangidwa liti?**  When was this latrine constructed? | ❒ **Chaka chino** (This year)  ❒ **Chaka chiyani** (What year) *Specify year: __ __ __ __*  ❒**Sindikudziwa (**Don’t know)  ❒**Akana (**Refused) | |
| **COMMUNICATION OF HEALTH MESSAGES** | | | |
| **60.** | **Kodi mumamverapo wailesi?**  Do you listen to the radio? | ❒ **Eya** (Yes)  ❒ **Ayi** (No) **→ GO TO 65**  ❒**Akana (**Refused)  **→ GO TO 65** | |
| **61.** | **Kodi mumamvera kangati wailesi pa sabata?**  *Mark only one.*  How many times in a week do you listen to the radio? | ❒ **Palibe** (None) **→ GO TO 65**  ❒ **Kamodzi pa mulungu** (Once a week)  ❒ **Kawiri kapenan katatu pa mulungu** (2-3 times a week)  ❒ **Kanayi kapena kasanu pa mulungu** (4-5 times a week)  ❒ **Kuposera kasanu pa mulungu** (More than 5 times a week)  ❒**Sindikudziwa (**Don’t know)  ❒**Akana (**Refused) | |
| **62.** | **Kodi ndi nthawi yanji imene mumakonda kumvera wailesi patsiku?**  *Do not read answers. Mark only one.*  What time of day do you most often listen to the radio? | ❒ **Mawa** (Morning)  ❒ **Masana** (Afternoon)  ❒ **Madzulo** (Evenings)  ❒ **Tsiku onse** (All day)  ❒ **Zimasinthasintha** (It varies)  ❒ **Ngati pali zina afotokoze** (Other, specify) __________________  ❒**Sindikudziwa (**Don’t know)  ❒**Akana (**Refused) | |
| **63.** | **Kodi wailesi yanu ya pa mtima imene mumakonda kumvera nthawi zambiri ndi iti?**  *Read answers. Mark only one.*  What is your favorite radio station? | ❒Seventh Day Adventist  ❒MBC 1  ❒MBC 2  ❒Capital FM  ❒Power 101  ❒Radio Maria  ❒Radio Islam  ❒Radio ABC  ❒ **Ngati pali zina afotokoze** (Other, specify)____________________  ❒**Sindikudziwa (**Don’t know)  ❒**Akana (**Refused) | |
| **64.** | **Kodi pologalamu ya pa mtima panu imene mumakonda kumvera nthawi zambiri ndi iti?**  *Read answers. Mark only one.*  What is your favorite program? | ❒*Youth Alert*! Mix  ❒**Nthawi ya Achinyamata**  ❒ **Kulankhula mwa tchutchutchu** (Straight Talk)  ❒ **Mauthenga a wana** (Kids Phone-in)  ❒**Pakachere**  ❒**Tikuferanji**  ❒ **Ngati pali zina afotokoze** (Other, specify)____________________  ❒**Sindikudziwa (**Don’t know)  ❒**Akana (**Refused) | |
| **65.** | **Ndi njira yanji yabwino yomwe mungamalandilire mauthenga aza ukhondo?**  *Read answers. Mark only one.*  What is the best way for you to receive health messages? | ❒**Wayilesi** (Radio)  ❒**Wayilesi ya kanema** (Television)  ❒**Munyuzi** (Newspaper)  ❒**Malo ophunzilira ana** (School)  ❒**Kukachisi** (Church)  ❒**Chipatala** (Hospital / clinic)  ❒**Kwa asinganga** (Traditional healer)  ❒**Pansonkhano** (Meeting with CHW / HSA)  ❒**Kwa amfumu / atsogoleri ena am’mudzi** (Message from chief /  other leaders in village)  ❒ **Ngati pali zina afotokoze** (Other, specify)____________________  ❒**Sindikudziwa (**Don’t know)  ❒**Akana (**Refused) | |
| **ADDITIONAL DEMOGRAPHIC INFORMATION** | | | |
| **66.** | **Kodi mumadziwa kulemba ndi kuwerenga?**  Can you read and write? | ❒ **Eya** (Yes)  ❒ **Ayi** (No)  ❒ **Akana** (Refused) | |
| **67.** | **Kodi sukulu munasiyira kalasi yanji?**  *Mark only one answer.*  What is the highest level of education you have attended? | ❒ **Palibe** (None)  ❒ **Pulayimale** (Some primary)  ❒ **Sekondale** (Some secondary)  ❒ **Ndinamaliza sekondale** (Completed secondary or higher)  ❒ **Akana** (Refused) | |
| **68.** | **Kodi pakhomo pano alipo ali ndi zinthu izi:**  *Mark all that apply.*  Do you or any member of your household own: | ❒ **Njinja ya kapalasa** (Bicycle)  ❒ **Njinga yamoto** (Motorcycle)  ❒ **Galimoto** (Car)  ❒ **Wayilesi** (Radio)  ❒ **Wayilesi ya kanema** (Television)  ❒ **Filiji** (Refrigerator)  ❒ **Magetsi a dzuwa** (Solar panel)  ❒ **Akana** (Refused) | |
| **CHLORINE TESTING** | | | |
| **69.** | **Kodi muli ndi botolo la Waterguard mnyumbamu?**  *Ask to see Waterguard bottle.*  Is there a bottle of WaterGuard in the house? | ❒ **Eya** (Yes)  ❒ **Ayi** (No)  ❒ **Akana** (Refused) | |
| **70.** | **Kodi madzi anu okumwa ali mbiya panopa ndi otetezedwa?**  Is the water currently in your stored drinking water container treated water? | ❒ **Eya** (Yes)  ❒ **Ayi** (No)  ❒ **Akana** (Refused) | |
| **71.** | **Kodi mungandigawireko madzi okumwa kuti ndiwone ngati mwathiramo kololini?**  ***Zitsatira zokhuzana ndi kulolini.***    May I take a sample of drinking water?  *Result of chlorine test.* | ❒ **Akana** (Refused) **→ END SURVEY**  ❒ **Palibe madzi** (No water stored) **→ END SURVEY**  ❒ **Mulibe** (Negative) **→ END SURVEY**  ❒ **Alimo** (Positive)  Free: ________ Total: ________ **→ END SURVEY**    ❒ **Pali zifukwa zina, lembani** (Other reason, if test not done):  **→ END SURVEY** | |
| **END SURVEY** | | | |

**Notes from the Household:**
